# A qualitative exploration of advantages and disadvantages to using technology in the process of randomised controlled trial recruitment

**DOI:** 10.12688/hrbopenres.13776.1

**Published:** 2023-09-14

**Authors:** Lauren A. Muldowney, Sinéad M. Hynes, Megan Oglesby, Christopher P. Dwyer

**Affiliations:** 1 School of Medicine, University of Galway, Galway, County Galway, Ireland; 2 Occupational Therapy, School of Health Sciences, University of Galway, Galway, County Galway, Ireland; 3 Teacher Education, Technological University of the Shannon Midlands Midwest, Athlone, Westmeath, Ireland

**Keywords:** trial recruitment, thematic analysis, trials methodology, patient and public involvement, technology in RCT recruitment

## Abstract

**Background:** Despite its importance, recruiting a sufficient sample size for randomised controlled trials (RCTs) can pose a significant challenge, which has real-world impact on reliability of evidence, trial completion and ultimately, patient care. Technology has potential to enhance the recruitment process, but there is a lack of evidence regarding its current use and effectiveness. Consistent with findings from the PRioRiTy I study, the current research aims to explore the advantages and disadvantages to using technology during the recruitment process for RCTs.

**Methods:** Semi-structured interviews (n=7) were conducted with researchers involved in RCT recruitment in Ireland. Subsequently, a Public & Patient Involvement (PPI) panel focus group (n=3) was conducted to add further depth to these findings. The data were qualitatively analysed through ‘Reflexive Thematic Analysis’ to extract prominent themes.

**Results:** A superordinate theme arose: ‘ *Tech is just a medium so that you can reach more people*’, along with two themes, which were corroborated by the PPI focus group: ‘ *Technology is used if and when the benefits outweigh the costs’* and ‘ *Success of recruitment through technology depends on the nature of the study*.’

**Conclusions:** This study provided a deeper understanding of the factors which influence researchers to employ technology in recruitment for RCTs. Implications suggest that future researchers should aim to adapt their recruitment approaches to meet digital tool preferences of their target cohort; and engage with patient groups in the community to allow networking opportunities for future studies. This research may contribute towards maximising efficiency in RCT recruitment.

## Introduction

Randomised controlled trials (RCTs) are widely considered to provide the highest level of evidence for an intervention’s efficacy ^1– 3^, with implications for participants and policymakers alike, making the optimisation of their efficiency and reliability a research priority ^4, 5^. A vital factor for ensuring the RCT success is achievement of an adequate sample size, as failure to do so increases the likelihood of type-II errors, trial extension and uncertainty surrounding what might be a potentially beneficial therapy ^6^. However, many trials do not achieve their desired sample size – just 56% of UK RCTs conducted between 2004 to 2016 achieved such targets ^7^. While this was an improvement on 1994-2002 rates ^8^, there remains a lack of understanding of optimal recruitment methods for RCTs.

RCT-participation is influenced by many variables, including perceived benefit, peer encouragement, the opportunity to help others and effective trial communication ^8– 10^. With respect to the latter, despite technological advances in communication methodologies and an ever-growing internet engagement (e.g. through social media), there remains a lack of research on the advantages and disadvantages of utilising technology in RCT recruitment – an issue which has been identified as an important research priority ^5^, given its potential to transcend more traditional trial communication methods and allow for a wider range of participants in RCTs.

Extant literature suggests that a considerable proportion of researchers may be using technology in some capacity in their clinical studies and along with its development, more opportunities to create and maintain effective contact with potential participants arise ^11^. Further to more traditional methods of recruitment (e.g., post, advertisement, and posters), a vast range of digital resources are available for use in RCT recruitment, from social media and direct-messaging to automated screening of health records and data mining. However, a disproportionate number of studies assess some technological methods (e.g. social media) as opposed to others ^12^. Moreover, as there is great variety in the types of technology researchers may use in recruitment, assessing the effectiveness of each may be challenging.

Though there is a limited body of research in this area, Rosa *et al*. ^13^ found that trial efficiency, lowered costs, enhanced stakeholder-involvement and reaching a more diverse range of participants are all advantages of using technology in RCT recruitment. Conversely, disadvantages may include privacy issues, inadequate infrastructure, exclusivity to particular populations and a lack of human interaction ^14^. In practice, however, there is little evidence of the true barriers and facilitators to using technology in RCT recruitment ^13^. Thus, the aim of the current study is to further explore the advantages and disadvantages of using technology in the RCT recruitment process and to facilitate deeper understanding of the pertinent issues. This may have future implications for the development of recruitment strategies for the benefit of both researchers and members of the public who are eligible to participate in such RCTs.

## Methods

### Ethical statement

This research was reviewed by and granted approval by the NUI Galway Research Ethics committee (Ref: 2021.05.007) on May 7 ^th^, 2021. Formal written consent was obtained by all research participants in this study.

### Study design

A series of one-to-one semi-structured interviews were conducted with consenting researchers who had previously been involved in the recruitment process for a RCT, with the aim of exploring perceived advantages and disadvantages of using technology during trial recruitment. An inductive, interpretive qualitative approach was used to explore participants’ perspectives, identify clear and relevant themes ^15^ and gain insight into participants’ experiences of topics lacking deep understanding ^16^. Following analysis of the interview data (Phase 1), a focus group was conducted with a *Public & Patient Involvement* (PPI) panel (i.e. people with lived experience of a particular condition as consultants throughout the research process ^17, 18^) in order to review the findings and further elaborate on concepts and themes, consistent with their experiences of being recruited for RCTs (Phase 2). As focus groups facilitate added depth of shared ideas through interactive discussion ^19, 20^, a PPI focus group was implemented in this manner to both add depth and richness to the analysis and interpretation of findings and provide a means of ensuring trustworthiness of the Phase 1 data ^9^. All data were collected and analysed via reflexive thematic analysis; thus, facilitating the inductive approach through an iterative, recursive process of identifying, analysing, forming and revising themes from the collected data ^21^.

### Materials

Zoom ^22^, a cloud-based videoconferencing application, was used to conduct and record the interviews and focus group. The semi-structured interview guide was developed in light of findings from the PRioRiTy I study ^5^ by a group of researchers experienced in the recruitment process (see Table 1). Questions within the guide were designed to both facilitate elaboration on experience(s) of using technology in recruitment, as well as to seek recommendations for its use in the future. Following its development, the interview guide was pilot-tested with the research team. In an effort to reduce bias, PPI members involved with the Phase 2 focus group were not included in the development or pilot-testing of the interview guide.

**Table 1.** Semi-Structured Interview Template.

1. What kind of recruitment strategies have you used where technology has been involved? (a) What kinds of technologies have you used in research recruitment? (b) Is there one technological method(s) you’d recommend over another?
2.Why was it decided to use technology to recruit?
3. Was there anything about using this technology that you found advantageous or facilitated recruitment?
4. Was there anything about using this technology that you found impeded recruitment?
5. Were you able to achieve the sample required? (a) If not, have you ever worked on study without technological support in recruitment where the required sample was achieved? (a.1) Why do you think that is? (b) Do you think using a recruitment strategy without this technology would have yielded a similarly sized sample?
6. What was your retention rate like for this RCT? (a) Do you think your recruitment approach impacted retention?
7. Can you describe the cohort you were seeking to recruit with regard to their age group, sex and educational or socioeconomic status? (a) Do you think that the type of cohort you were recruiting (e.g. with respect to age, gender, ethnicity, socioeconomic status had an impact on your recruitment process)?
8. Did the use of technology cost anything above and beyond that of not using it with respect to resources? (a) Do you think it was worth these costs?
9. Has COVID impacted the way in which you *think about* recruiting for RCTs?
10. How might technology be used to improve recruitment for randomised controlled trials in the future? (a) What do you think of the real-world implications of such recommendations (e.g. with respect to feasibility, accessibility and appropriateness).
11. Do you think there are any other potential barriers to using technology for recruitment that have not yet been discussed?
12. Do you think there are any other potential factors that can enable recruitment that have not yet been discussed? (a) Any final thoughts/comments

### Procedure

Previously funded RCTs, conducted in Ireland since 2006, were identified by database searches of Ireland’s four major funding bodies. Direct contact was then made to researchers on 50 identified trials, inviting participation via email. In addition, a descriptive flyer was circulated on social media using Twitter, Facebook and LinkedIn, with a contact email made available for interested parties. Potential participants were provided with information about the study in the email, as well as a participant information sheet and consent form. The information sheet was also forwarded to eight clinical research facilities, based in Ireland, for further circulation.

Eight individuals expressed interest in participating in an interview, one of whom was unable to take part due to scheduling issues. All of the remaining seven (N = 7) individuals were eligible according to the criteria that the individual was a consenting (1) researcher (2) previously involved in RCT recruitment, (3) based in Ireland; and participated in the virtual audio-recorded interviews (conducted between July-August 2021; mean duration 28 mins). Data were coded, analysed and themes identified (Phase 1) ^1^.

In Phase 2, a PPI focus group (N= 3; duration 52 mins) was conducted in light of findings from the previous phase, in order to further explore and elaborate on concepts and themes. PPI panel members were first presented the semi-structured interview questions asked of the interviewees as a means of introducing the concepts of interest and to further explore consistency between perceptions of the panel (i.e. former/potential RCT participants) and then actual Phase 1 responses, particularly with respect to advantages and disadvantages of using technology in the recruitment process. The PPI panel was asked the questions prior to being advised of Phase 1 responses and themes, in order to limit any potential bias.

### Data analysis

Transcribed data were checked by two researchers for context and errors. Microsoft Office was used. Consistent with Braun and Clarke ^23^, (1) data familiarisation began during data collection and involved the reading and re-reading of the interview transcripts, accompanied by observational note-taking; (2) researchers then systematically generated concise, meaningful codes; (3) identification of patterns from/within the data, both of which were discussed by the researchers; and (4) themes identification; prior to completion of the iterative review and refinement of these themes against the transcripts, for the purpose of ensuring their credibility ^24^. Thus, trustworthiness of the data and credibility of the findings were ensured in multiple ways, such as triangulation (e.g. multiple observers/observations and analysts/analyses), researcher immersion in the data for ensuring rich descriptions, as well as consultation of the PPI panel.

## Results

Table 2 presents relevant demographic information for each interview participant (N=7; 6f, 1m) ^25^. PPI panel members (N=3; 1f, 2m) consisted of individuals living with a chronic illness who have either taken part or have been previously eligible to participate in a RCT relevant to their illness. Pseudonyms were used for all participants in this study.

**Table 2.** Demographic information.

Pseudonym	Research Level	Trial Design	Field of Study	Participant Age Group	Technology Used
*Anne*	Post-Doc	Pilot RCT	Endocrinology	Young Adults (18–25)	• Phone Calls • Postal service
*Beatrice*	Senior Lecturer	Pilot RCT	General Practice	Not specified (18+)	• Emails • Mailing Lists • Social Media (Twitter)
*Craig*	Post-Doc	RCT	Mental Health	School-age children	• Emails • Phone calls • Participant Recruitment Websites
*Deirdre*	PI	RCT	Mental Health	Older adults (65+)	• Social Media (Facebook, Twitter)
*Eleanor*	PhD	RCT	Neurology	Not specified (18+)	• Videoconferencing (Zoom) • Phone Calls
*Frances*	Lecturer	RCT	Breast Cancer	Not specified (18+)	• Phone Calls • Videoconferencing • Emails
*Grace*	Clinical Research Coordinator	RCT	Intensive Care	Not specified (18+)	• Phone Calls

### Phase 1

**Figure 1.**
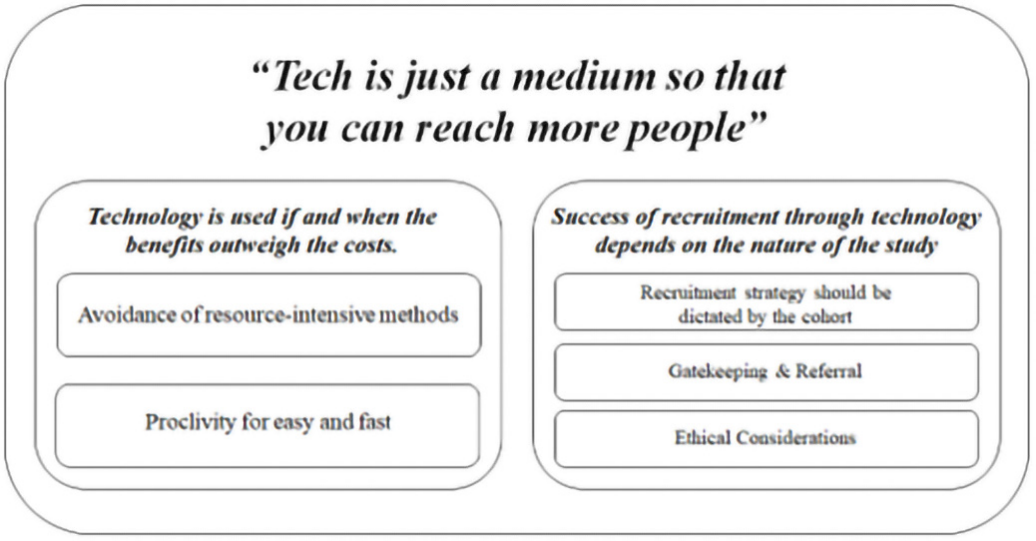
Outline of themes identified.

Consistent with the aim of this study, a number of advantages and disadvantages of using technology in the RCT recruitment process were identified, as presented in Table 3. With respect to the study’s other aim – that is, to facilitate deeper understanding of the issues pertinent to using technology in the recruitment process, findings from the reflexive thematic analysis identified one over-arching, super-ordinate theme; two themes and five sub-themes (see Figure 1).

**Table 3.** Advantages and disadvantages of using technology in the RCT recruitment process.

Medium	Advantages	Disadvantages
***Overall Use of Technology***	• Can be less resource-intensive • Allows more targeted recruitment on a larger scale • Preferable to face-to-face contact in certain contexts	• Participants may still want to meet in person • Requires IT skills of both participant and researcher • Data security considerations • Issue of representativeness
***Phone Calls***	• Quick, direct way to contact potential participants and check their status in the ‘sign-up process’ • More personal	• Requires initial contact by other means be it clinical or otherwise
***Emails***	• Can attach relevant documents (e.g. information leaflets) • Cheap, fast • Allows reminders to be sent • Recipient has more agency about when to engage with the information	• Less personal and thus easier to ignore • Cold-emailing tends to be less successful than when a relationship has been established/a gatekeeper is used • Requires more IT skills than traditional phone calls or post
***Post***	• May be preferable to participants who like to physically read information on paper	• Less frequently used nowadays, especially for younger people. Many unfamiliar with going to post office • Relatively cheap • Slower
***Videoconferencing***	• Most effectively simulates a face-to-face meeting	• Platforms are of variable quality • Some IT skills required
***Social Media***	• Quick and cheap • Allows widespread communication to large numbers of people • Specific social media sites can be utilised according to demographics desired	• Needs to be shared by those with larger following to reach required participants • Can be too widespread, difficult to reach specific populations • May exclude certain cohorts e.g., older people who may be less engaged with social media • Less room for information – character limits etc. • Can be considered less credible in some cases
***Mailing Lists***	• Allows researcher to target a very specific desired population • Those signed up are more likely to be interested in research	• Require trust/relationship with a relevant body to allow access to mailing list
***Participant Recruitment *** ***Websites***	• Monetary incentives are provided for participants • Can recruit large numbers in short space of time	• Expensive for researchers and create disadvantage among researchers who cannot afford to use them

Super-ordinate theme: *“Tech is just a medium so that you can reach more people”*

The overarching theme – or message – of the findings was that the use of technology in recruitment is a means of reaching as many people as possible to ensure the sample necessary for the study. If a certain technology could facilitate that and was feasible to utilise, then it would be engaged. This superordinate theme is further explored throughout the analysis regarding the themes and sub-themes.

Theme 1: *Technology is used if and when the benefits outweigh the costs*.

The first theme addresses how technology is utilised to facilitate recruitment when its use is perceived as convenient and inexpensive with respect to financial cost, time and other resources to both researchers and patients. Data from the interviews suggest that technology was not used just for the sake of using it, but with the intention of meeting logistical needs of a particular study’s recruitment process.

*“I'm a little bit wary when people say that they're going to use technology as a way around things, whereas technology, I think, is a facilitator for things that are going to work or not work in the first place” - Craig*

This is a key finding of the study, which emphasises the utility of technology as a tool that has potential to enhance the recruitment process but cannot necessarily eliminate all existing barriers. Use of technology must be relevant in the context of meeting an unmet need in research recruitment and should never make this process more difficult.

Within this theme, two further subthemes were identified (i.e. ‘ *Avoidance of resource-intensive methods’, ‘Proclivity for easy and fast’*). The tendency towards avoiding ‘ *resource-intensive methods’* was noted throughout the interviews. Cost and budget considerations tended to influence what was feasible in the recruitment process. The cost of purchasing a new technology is cited as a barrier to its use, as recruitment may not be the first priority in the allocation of funds. Likewise, cost to the potential participant and its impact on the decision to take part was also an important consideration. For example:

*“You wouldn't often think about allocating a lot of resources to the recruitment strategies. …We'd be relying on existing networks… If it's something that takes a lot more on their part, I'd be less inclined to use it… it comes down to the time and cost for the participants and how likely it is that they're going to engage with us.” - Beatrice*

Aside from monetary cost, time was a resource impacted by the choice of recruitment method. The use of technology to increase reach, with respect to advertising recruitment, saves time in the recruitment process. Where a large sample of participants is required in a timely manner, technological methods can provide a quick means to access the patient cohort needed:

*“Time-wise, I think, casting a wider net - which I could do with tech – it saved me time compared to… recruiting much more locally, where you're dependent on a small few eligible samples, where the hit rate is much lower.” - Craig*

*‘Proclivity for easy and fast’* was the second subtheme, which captured the essence of technology, with respect to its general purpose and strength of meeting the needs of the research study. Thus, the identification of this theme is somewhat unsurprising. The use of existing technological resources for the purpose of convenience became evident throughout the interviews – essentially, ‘use what’s there’:

*“Just think about the budget you will need. If you’re going to do things virtually… we just used the resources that we have. Your sponsor can just have… already… some platforms and nowadays with video calls, it's like all the companies have some sort of platform already arranged.” - Frances*

Despite budgetary concerns surrounding use of technology, less resource-intensive ‘technological’ methods were utilised by researchers (e.g. phone calls and emails). These were described as a cheap and convenient means for contacting interested parties:

*“Because it was email, I suppose, it's not something that we needed to train ourselves. Skills–wise, we could do it; time-wise, we could do it; and it didn't cost anything, financially, or in terms of time. So, I don't think it was a bigger drain on… it's very low resource way for me.” - Beatrice*

Use of technology may also facilitate ease with respect to reducing the time that prospective participants may have to spend in face-to-face environments. Technology can be used to minimise such contact, while simultaneously reducing travel costs. For example, use of videoconferencing allowed some researchers to meet with potential participants and engage in the informed consent process virtually:

*“It's more comfortable for the patient – just doing this step online; they are going to come one more time to the clinic for the baseline. But, at least, they don't need to come for the informed consent and they can just be comfortable at home… not having to drive here.” – Frances*

This concept also has consequences for the ‘scalability’ of technology, which potentially allows for a larger pool of participants to be reached, thus making technological methods more convenient in comparison to traditional methods in large-scale studies. Where a specific target population is required, technology may just be ‘easier’:

*“Whether we would have got the spread [without technology]… I don't think we would have. We would have been relying more on a convenience sample. So, I definitely think that this gave us the scale to reach more practices.” – Beatrice*

*Theme 2: Success of recruitment through technology depends on the nature of the study*

The second theme describes the variability of recruitment methods required, depending on the type of study being conducted. It acknowledges the vast differences among individuals and their preferences for engagement with a research study. Three subthemes were also identified: *recruitment strategy should be dictated by the cohort; gatekeeping and referrals; and ethical considerations.*

It was clearly recognised throughout the interviews that the ‘ *recruitment strategy should be dictated by the cohort*.’ This subtheme recognises the diversity among potential participants and, consequently, their variable engagement with technology. Age featured as a point of comparison regarding levels of engagement with various means of communication. For example, it was suggested that more mature cohorts might prefer less technologically advanced methods communication (e.g. mail/post), whereas younger cohorts more technologically advanced methods (e.g. email, text and video-calls). According to Grace:

*“It's the older generation versus the younger generation; and the older generation is what we still see a lot of in our cohort of patients. So, even though we have emails to say it to some people… they like the written printed-out version”*

The preferred method of communication, be it through advertising the study, providing an information sheet or even obtaining informed consent, is an important point of consideration when targeting a specific group of participants. Similarly, the variability of IT skills among potential participants should also be considered. For example, when technology is a feature, there are often some participants who require more support in accessing or using the technology:

*“One of the initial problems was the technology itself and technical issues about setting up, but then usually their children or grandchildren will be there to set them up with the video call – but then if they’re alone, we wouldn't get a lot of inputs out of them.” – Eleanor*

This reflects an important consideration regarding the first theme – while it is preferable to choose an efficient method with respect to resources, ease and speed, the recruitment method also needs to be accessible to the target cohort or it will, inevitably, fail to recruit the desired cohort.

Even though technology can be a barrier for some potential participants, if there is a will, there is often a way with respect to finding the supports needed to engage the technology (such as in Grace’s example of having aid from family members). However, it must also be acknowledged that support is not always feasible. Thus, technology is a hurdle to some. 

Whereas thinking and discussion often revolved around the level or type of technology used in the recruitment process, the manner in which recruitment was *targeted* was also an important factor. Largely consistent with the super-ordinate theme that *‘tech is just a medium so that you can reach more people’,* the concept of targeted advertisement is vital for considering how best to reach a *particular* audience, especially if the required cohort is ‘specific’ or ‘niche’. In this context, the interviews suggested that regardless of whether or not ‘technology’ is utilised as a means for recruitment, the method is only useful if it reaches the cohort suitable for the study:

*“Social media feeds and stuff are all based on how much somebody's using them – if if someone's sharing it and they've only got 20 people on it, that’s a disadvantage. It's more advantageous if it's picked up by somebody with a high [number] of followers … Then the advantage is there; especially… if any of the [health service] national groups pick it up and forward it on, you get much better reach. So, it's really dependent on who's actually posting it, whose news feed and how it then gets marketed; and that's the thing as clinicians, we aren't taught marketing… and that's what you're basically asked to do.” - Deirdre*

Notably, just as the cohort *should* dictate the recruitment method, the recruitment method *could* potentially dictate the cohort. As alluded to above, the way recruitment is advertised and conducted could impact the diversity or representativeness of the sample, which might be otherwise unaccounted for within the recruitment design.

*“Depending on how [technology’s]used, you may only get the people who are very engaged; so, if it's something like Twitter or social media, thinking about how representative those people are and how equal is it for everyone to be able to engage with that technology.” - Beatrice*

The second subtheme, *‘Gatekeeping & Referrals’,* addressed the concept of a ‘gatekeeper’ and their utility in research recruitment. The term was aptly defined by one of the researchers interviewed:

*“It’s even more integral now with GDPR because you can't access people's information... so, a gatekeeper is a person who has access to that information and can share your study information with people, but they're not… abusing their access to the information – they’re not pressuring the person.” - Beatrice*

This concept emerged across the interviews and illustrated a sense of trust that aided study participation. Providing information about a study via a trusted individual or organisation (e.g. study advertisement or a participant information sheet) was perceived as a successful strategy:

*“I think having a credible source be the messenger for your study is really valuable. Whether that also involves technology, that's totally possible; but, I think having other GPs endorse my study or having them kind of promote the study or suggest it to their colleagues carries more weight than me trying to do so.”- Beatrice*

This sub-theme also suggests that relationships and networking similarly provided a solid foundation for researchers to gain access to recruit from particular groups, irrespective of using technology:

*“It was a little bit of a nightmare…And then the only reason that I was able to recruit… after that is because I knew somebody who knew somebody.” - Craig*

The final subtheme elicited was *ethical considerations*, which encompassed the systemic structures (e.g. committees) underpinning ethical research as well as the increasing concern for data security within society. Research ethics committees, while crucial for maintaining the safety of participants and integrity of research, emerged as a source of frustration for researchers:

*“The recruitment strategies were… it's slightly different in each hospital, because the ethics committees required different strategies. So that was a bit frustrating, but we had to do it.” - Anne*

Likewise, ethical approval requirements were perceived as a barrier to recruitment:

*“The more interesting the sample, the more difficult it was to access them and I suspect that's the same in other areas too. It certainly complicated recruitment further. So, I honestly think that the ethics committees, generally speaking, make it unnecessarily harder. I know that there are certain laws that they have to abide by and certain principles, but I find that they're not very pragmatic and that can hinder recruitment.” - Craig*

Notably, it can be argued that this quote also relates back to the concept of ‘recruitment being dictated by the cohort, in the sense that ‘ *the more interesting the sample, the more difficult it was to access them’* suggests that specific populations that may be the target of a study may require a different strategy for recruitment – less so an issue of whether technology would or would not be advantageous, rather an issue of ethical consideration – particularly, if the ‘interesting sample’ was deemed vulnerable. 

 Notwithstanding the obstacles encountered in gaining ethical approval, data security was recognised as a vital aspect of research and recruitment. Respect for participant data and acknowledgement of the trust participants have in researchers to protect data responsibly was evident among the interview data:

*“There should always be a backup, so that you can protect the integrity of the study and the integrity of the data that you're collecting… for the patients’ privacy... We need to protect their data.” – Grace*

Data protection and security is a matter of public concern that has gained considerable attention in recent years. Trust is required in the recruitment process for patients to consent to sharing personal and often sensitive data for the purposes of research. Clarity should be provided when enrolling participants to a trial with regard to how their information will be collected, stored and used. This is of utmost significance in the context of technological methods of recruitment, both in the sense of how data are collected securely at this early stage of a trial and in setting the precedent for further trial communication. Robust processes need to be demonstrated to avoid distrust, which could influence retention and completion of trial enrolment.

### Phase 2

Following presentation of the interview guide to the PPI panel and subsequent discussion of interview results, the PPI focus group findings were largely consistent with those of Phase 1 and provided further perspective and depth to the identified themes. The first theme, *Technology is used if and when the benefits outweigh the costs,* was addressed by the panel, who agreed that technology should be used when it is convenient and when the cost is acceptable. In this respect, the panel paid particular focus to emphasising that *Avoidance of resource-intensive methods* should, as recommended in Phase 1, extend to consideration of potential participants’ resources (be it time, financial or otherwise), as recruitment strategies can either limit cost or be costly. For example: –

*“And like I live over in [county] and I was driving over 45 miles over to Galway .... If I could have done it over the web, like, it’d be great.” – Calum*

Likewise, according to Ben:

*“You'll have a certain cohort of people that, say they've lost time from work or they've lost time from their family... If you have somebody that has chronic fatigue, every hour that you have to do something, that's precious. So, you're going to focus on your family or you're going to focus on your kids, rather than on research. So, how do you compensate for that?”*

Thus, in order for recruitment to be successful, methods of accommodating participants to engage with research should be understood and engaged by the research team – ultimately influencing both recruitment and study design.

 The concept of *‘Proclivity for easy and fast’*, was understood by the panel to mean that researchers choose the method which is easiest and fastest for the research team. For example, by choosing tools that are already accessible via their institution rather than exploring other options:

*“I’m at the stage now where if I was told by my consultant that I was going to be called on [specific videoconferencing platform] I would say, ’Do you know what? I’m fine. I’ll wait until I can come and see you in person' – I can’t be doing this. It’s exhausting – especially if you have any kind of extra ability issues going on at the same time. It’s just – no.” – Arlene*

Like researchers, patients exhibit a preference for recruitment methods that are convenient and least disruptive to their lives. However, these methods may not always be congruent between researchers and their desired sample, which is consistent with discussion of the second theme, *‘Success of recruitment through technology depends on the nature of the study’* – particularly, the sub-theme of ‘ *recruitment strategy should be dictated by the cohort*’. For example:

*“I think that whoever is designing whatever trial – they need to know what their patient cohort uses, not what they use.” – Ben*

In a cumulative sense, it must also be acknowledged that, through presentation of the subtheme regarding *ease* and *speed*, there may be the assumption by the PPI that researchers only consider these issues from their own perspective, where it could well be the case that the researcher also thinks that such a method would facilitate potential participants. Though the suggestion of uniformity may not necessarily facilitate a ‘happy medium’ between researchers and potential participants, the concept of ‘uniformity’ introduced here may more so refer to the need for researchers and potential participants to ‘get on the same page’ – in terms of congruency, with respect to what methods work best and for who, as addressed above. Thus, it may be the case that a mixed-approach for utilising technology, via recruitment through multiple avenues, might work best in this context. Such a mixed-approach for designing the recruitment strategies was favourable among the panel, for example, in recognition of the natural heterogeneity of individual patients and patient groups:

*“I think you just need to have a balance. I think you can't go one way or the other… if you want to include everybody or try and hit as many people as you can.”– Arlene*

Consistent with this diversity of patient preference, the panel described methods which they themselves preferred, with some having a tendency towards the written word (sometimes facilitated through technology), while others appreciated the growing opportunities technology provides. According to Calum:

*“I suppose like a few paragraphs… text-based stuff… so you can read it – something physical, tangible even if it is a PDF document – and you can kind of mull over it and you know [researcher]’s email address was in it. So that's the way I’m quite happy working with.”*

According to Ben:

*“If I have to go somewhere, it's harder and harder to leave these four walls. It's incredible, what’s happened now is that I can access stuff that’s in Switzerland and the United States... I'm not geographically bound anymore, which is incredibly liberating.”*

This issue of diversity is additionally interesting to consider because even when putting participants before the ease, speed cost or even preferences of the researcher(s), there remains no guarantee that all individuals within a targeted cohort will prefer the same means of communication as others. Levels of engagement with each medium will vary, as will access to technology and infrastructure, regardless of the cohort or, in the context of the PPI focus group, patient group one belongs to. This point may also have some implications for the likelihood of finding uniformity (as addressed above) between researchers and potential participants. 

Moving forward, relationships and networking were also emphasised as being integral to accessing a desired patient group. Similar to the *‘gatekeeping and referrals’* subtheme, value was placed on having a trusted person or body to connect the patient with the researcher:

*“We have a forum that does a whole lot of wonderful things locally and they have very tight networks, so, if you have access…to one person – then they can spread it throughout their network very quickly, and that will aid your recruitment.” – Ben*

Likewise, according to Calum:

*“So long as you’ve someone trustworthy… It’s not just some kind of random email you open up that could be God knows what.” - Calum*

Upon delving further into the feasibility of networking, the contrast between the worlds of researchers and participants emerged as a potential barrier to accessing local groups. The PPI panel felt that both entities seem to occupy different spaces, which can make the process of forging connections between the two more difficult:

*“Researchers live in their bubble and everyone else isn’t in their bubble… which is quite unusual because we need each other. We need the researchers, just as much as they need us; and as patients, we want to hear from them. We want to be talking to them. We would like to be influencing the studies that they’re doing; yet, they don't seem to have access… researchers need to get out of their offices and… they need to be part of that community.”” – Ben*

Nonetheless, the panel was also understanding of researchers’ perspectives, acknowledging that ethical considerations from Phase 1 findings may also play a role in their decision-making – arising as another potential barrier to networking. This is an aspect of research culture the panel seemed to feel was old-fashioned, which could be improved upon to aid recruitment. Likewise, consistent with the dynamic nature of technology, recruitment strategies evolve over time; and so, there may be a need to shift the status quo within research to keep up with this evolution. This panel viewed such a shift as a significant challenge:

*“I think there's a fear amongst researchers that they will be doing something that's unethical... There's a reluctance amongst some people to change the way they've done it historically, ‘this is the way we've always done it, and this is the way we've done our trial’ and to change that mindset is a real challenge.” - Ben*

## Discussion

### Interpretation of results

Upon analysis of Phase 1 data, one overarching theme was identified: ‘ *Tech is just a medium so that you can reach more people’*. This represents the concept that the utility of technology lies predominantly in its ability to scale up the recruitment process and may not require any great consideration beyond this utility, except for issues relating to cost and the nature of the study itself. Notably, the term ‘technology’ was presented to participants in a purposefully vague manner, so as to not bias their views. It was hoped that some would endeavour to provide their definition to better conceptualise technology in this context. Though no such definition was provided, a series of different technologies were discussed – mostly in terms of web-based applications (e.g. email, social media, text-messaging and video-calls). Phone calls and post/mail were also discussed, but not necessarily in terms of ‘technology’.

Herein lies an interesting consideration – technology evolves over time. In research 60 years ago, the telephone might be considered among peak technologies. Before that, post/mail might be considered as such as well. Indeed, something as simple as the printed word was once considered technology. Just because time has elapsed and technology evolved does not mean that these means of communication have ceased to be ‘technology’; for example, given that people still use post/mail and the telephone to communicate. In the results it was found that some people prefer these older technologies. Thus, if the aim of recruitment is to reach as many people as possible to achieve one’s sample size, the age or format of a technology does not matter, just if it remains relevant and useful for achieving the necessary sample.

 Two central themes were addressed within the superordinate. The first, ‘ *Technology is used if and when the benefits outweigh the costs’,* presents the idea that availability of resources impact how technology is implemented to recruit for RCTs and also how researchers take preference for the method which is easiest and fastest for both themselves and the potential participants. The second, ‘ *Success of recruitment through technology depends on the nature of the study’,* addressed the contrast between different study designs and procedures, as well as how this influences the recruitment process. Within this theme, the variation in participants’ engagement with technology, the pivotal role gatekeepers and referrals play in successful recruitment and the impact of ethical considerations on recruitment were also discussed. In summary, choosing a successful method(s) for recruitment requires consideration of the specific characteristics of the target cohort; and establishing networks that link participants and researchers is often vital, regardless of ‘technology’.

Phase 2 data corroborated these findings, while also shedding light on the results from a patient perspective. The panel found value in the themes’ message and re-emphasised that resource considerations should extend to the cost a potential participant undertakes as a research participant. The necessity for a culture shift in research was also highlighted to bridge the communication gap between researchers and patients. Moreover, the heterogeneity of individual patients within a group may influence their engagement with a specific technology or method of communication. Willingness to incorporate multiple methods to reach a larger subset of desired patient groups may be a key step in combating this issue.

 The findings highlight a number of similarities and contrasts with extant, though limited research. Of note, the current research was particularly consistent with the qualitative portion of a study by Blatch-Jones *et al*. ^14^ which argues that the availability and acceptability of strategies across different patient groups requires consideration as does the trade-off between utilising digital strategies and more personal interaction.

 Regarding the common use of specific technology, participants mentioned phone calls, emails, social media, participant recruitment websites, post, videoconferencing and mailing lists, which were consistent with a recent review by Frampton *et al*. ^12^ of 105 RCTs. While studies have cited use of an electronic health record to allow automated identification of potential participants ^14^, this was not an approach that arose from our study – perhaps due to incompatibility of current electronic health records for this type of use in the Irish context. With that, extant literature has not been able to comprehensively compare the efficacy of individual methods of digital recruitment; instead, focusing on particular methods in isolation, such as social media ^26^. While two participants in the current research noted the utility of social media in reaching a larger number of widely distributed potential participants, the need for a link or ‘gatekeeper’ to successfully aid this method was also emphasised.

### Limitations

Though a number of interesting findings were yielded, some specific limitations must be addressed. First, the sample size can be argued to be small. Overall, eight individuals expressed interest in participating, in which the irony of the situation is acknowledged – wherein there was difficulty in recruiting for a study on recruitment. Nevertheless, the data yielded from this ‘small’ sample was qualitatively rich; and the sample attained is consistent with recent research ^9^ which utilised a similar dual-phase approach with one-to-one interviews conducted prior to a corroboratory focus group. Moreover, sample size in qualitative research is a subjective consideration – the priority should be achieving a sample that is not too large that it obstructs the process of deep analysis, consistent with the notion that the more relevant information available within the sample, the lower the number of participants needed ^27, 28^. Thus, in the current research, it is reasonably argued that the sample size is justified on account of the richness and extensive analysis of the available qualitative data.

 Furthermore, as addressed above, a standardised definition of technology was not presented to interviewees, which allowed for a wider interpretation of ‘technology’ in this study. It is possible that this limited some level of comparability between interviewees’ responses. However, consistent with discussion regarding the super-ordinate theme identified, it can also be appreciated how this added depth in allowing each researcher to individually contemplate what technology means, in general, as well as in the context of their own trial.

## Conclusion

The results of this study contribute a deeper understanding of the pertinent issues for using technology in recruiting potential participants for RCTs. Key factors that influence the choice to use technology were highlighted (e.g. cost, ease, speed/time and the nature of the study), with particular emphasis on technology being no more than a medium to achieve research aims. The need to adapt recruitment design to suit target cohorts is of significance. The current research arguably succeeded in answering Healy *et al*.’s ^5^ PrioRiTy question 10: *‘what are the advantages and disadvantages to using technology during the recruitment process?’*, through providing a thorough list (again, Table 3) and elaborating on technology as, simply, a medium for communication and both advantages and disadvantages to its use as contextual –likely to be utilised when easy, fast, lacking resource-intensiveness and consistent with the nature of the study.

 In keeping with the qualitative nature of this study, the findings cannot be generalised to larger research populations; however, they have facilitated deeper understanding of the advantages and disadvantages of using technology in the RCT recruitment and other pertinent issues. Such understanding provides a useful starting point for further research to investigate the themes identified – for example, future research should aim to: adapt their recruitment approach to acknowledge the preferences and costs to potential participants (regardless of how ‘technologically advanced’ it may be) and engage with potential participants to facilitate networking opportunities for future studies. Further research into the use of technology in RCT recruitment is warranted, particularly surrounding its effectiveness when used in multiple ways or in combination with more traditional methods. In light of these findings, the identified advantages and disadvantages of using technology for RCT recruitment can be appreciated, while also acknowledging that the use of technology is a multifaceted decision in this process, integrating resource considerations, study design issues, as well as potential participant preferences.

## Data Availability

Open Science Framework: Advantages and disadvantages to using technology in the process of randomised controlled trial recruitment. https://doi.org/10.17605/OSF.IO/67GM5 ^25^. This project contains the following underlying data: Focus Group anonymised final.docx Interviews anonymised.docx Data are available under the terms of the Creative Commons Attribution 4.0 International license (CC-BY 4.0).
