# Supplementary material for: A qualitative exploration of advantages and disadvantages to using technology in the process of randomised controlled trial recruitment
Source: HRB Open Res. 2024 Jun 6;6:56. Originally published 2023 Sep 14. [Version 2] doi: 10.12688/hrbopenres.13776.2 (PMC11358689; doi:10.12688/hrbopenres.13776.2)
Supplement: Peer review history

# Reviewer report: Reviewer response for version 2

**DOI:** 10.21956/hrbopenres.15257.r41763

**Published:** 2024-08-28

**Authors:** Milan Bimali

**Affiliations:** 1 University of Arkansas for Medical Sciences, Little Rock, USA

Randomized controlled trials are considered “gold standard” in biomedical research. One of the reasons for failure of RCTs is their inability to accrue study participants. This manuscript covers an important aspect related to recruitment. It focuses on the use of technology in the context of participant recruitment in RCTs. This is important because the use of technology is becomingly increasingly inevitable and its application in recruitment in RCTs can be an important topic for scientific community. The manuscript is based on a qualitative approach and makes use of structured interviews with researchers involved in RCT while drawing on their perspective and opinions related to the use of technology in RCT recruitment. The analysis was done using the “Reflexive Thematic” method to identify key themes. The authors identified two themes – technology is used if and when benefits outweigh the costs; recruitment success through technology depends on the nature of the study.

 I have included below my review comments:

1. The authors has consistently the term “adequate sample size” throughout the manuscript. The term “adequate” appears very subjective and vague. Often RCTs are powered and the sample size is statistically justified. In some studies, the sample size is justified based on precision estimates. The authors must reconsider the use of term “adequate”.
2. The authors have used the “reflexive thematic analysis”. I would suggest adding a brief description about it, so readers have a basic understanding of it.
3. The “Introduction” section must recognize that as of now, not all RCTs have the option of incorporating technology in recruitment. This may be especially true for studies in resource limited setting where technology is not available.
4. The limited sample size in this study has been acknowledged by the author. This has greatly limited the generalizability of study findings. It is also unclear whether there was a self-selection bias at play here. Was there a systematic difference in the fundamental characteristics of studies whose investigator agreed to participate in the study?
5. It would also be helpful if the authors delved a bit more into either the introduction or discussion on the ethical issues related to use of technology in recruitment.

Is the work clearly and accurately presented and does it cite the current literature?

Yes

If applicable, is the statistical analysis and its interpretation appropriate?

Not applicable

Are all the source data underlying the results available to ensure full reproducibility?

Yes

Is the study design appropriate and is the work technically sound?

Partly

Are the conclusions drawn adequately supported by the results?

Yes

Are sufficient details of methods and analysis provided to allow replication by others?

Yes

Reviewer Expertise:

Biostatistics

I confirm that I have read this submission and believe that I have an appropriate level of expertise to confirm that it is of an acceptable scientific standard.

# Reviewer report: Reviewer response for version 2

**DOI:** 10.21956/hrbopenres.15257.r40525

**Published:** 2024-06-08

**Authors:** Marie Falahee

**Affiliations:** 1 University of Birmingham, Birmingham, England, UK

Thank you for addressing my comments.

Is the work clearly and accurately presented and does it cite the current literature?

Yes

If applicable, is the statistical analysis and its interpretation appropriate?

Not applicable

Are all the source data underlying the results available to ensure full reproducibility?

Yes

Is the study design appropriate and is the work technically sound?

Partly

Are the conclusions drawn adequately supported by the results?

Yes

Are sufficient details of methods and analysis provided to allow replication by others?

Partly

Reviewer Expertise:

Psychology, Rheumatology, Patient and Public Involvement in Research

I confirm that I have read this submission and believe that I have an appropriate level of expertise to confirm that it is of an acceptable scientific standard.

# Reviewer report: Reviewer response for version 1

**DOI:** 10.21956/hrbopenres.15069.r36770

**Published:** 2024-04-30

**Authors:** Upasana Baruah

**Affiliations:** 1 National Ageing Research Institute, Victoria, Australia

This research paper explores the role of technology in the recruitment process for Randomized Controlled Trials (RCTs) by conducting a dual-phase qualitative study involving interviews and a patient and public involvement (PPI) focus group. The study reveals an overarching theme that perceives technology as a medium to broaden participant reach. The findings emphasize the importance of considering resource costs, study characteristics, and participant preferences when incorporating technology into recruitment strategies. The discussion delves into the evolving nature of technology, addresses central themes related to cost-benefit analysis and study-specific recruitment success, and effectively integrates patient perspectives from the PPI focus group. The conclusion summarizes key findings, highlights the study's contribution to understanding technology's role in RCT recruitment, acknowledges limitations, and suggests avenues for future research, providing a comprehensive overview of the nuanced considerations surrounding the utilization of technology in clinical trial recruitment.

 While the paper adeptly explores the role of technology in RCT recruitment through a well-executed dual-phase qualitative study, a noteworthy limitation lies in the small sample size, particularly in the PPI focus group where data saturation becomes challenging with only three participants. The authors acknowledge this limitation, but clarity on the rationale for this specific number is lacking.

 Furthermore, while the recruitment process is adequately detailed, inclusion of participant characteristics, such as gender, experience, or roles in recruitment, would enhance transparency and generalizability.

 The paper mentions using Microsoft Word for analysis without specifying the process, necessitating clarification on whether any qualitative software was employed for data management and analysis. The data management and safety plan require elaboration for a comprehensive understanding.

 The results section might benefit from a more succinct presentation of main themes and sub-themes, avoiding exhaustive detail.

 Additionally, the initial paragraphs of the discussion seem repetitive of the results and could be condensed to emphasize key discussion points more concisely.

Is the work clearly and accurately presented and does it cite the current literature?

Yes

If applicable, is the statistical analysis and its interpretation appropriate?

Yes

Are all the source data underlying the results available to ensure full reproducibility?

Yes

Is the study design appropriate and is the work technically sound?

Yes

Are the conclusions drawn adequately supported by the results?

Yes

Are sufficient details of methods and analysis provided to allow replication by others?

Partly

Reviewer Expertise:

dementia, carer support interventions, psycho-social interventions delivered remotely, mental health

I confirm that I have read this submission and believe that I have an appropriate level of expertise to confirm that it is of an acceptable scientific standard, however I have significant reservations, as outlined above.

# Response

**Published:** 2024-05-29

**Authors:** Christopher Dwyer

**Affiliations:** Technological University of the Shannon Midlands Midwest, Athlone, Westmeath, Ireland

Many thanks for your comments. We considered each in turn and have amended the manuscript in light of these considerations. Other comments and questions we answer here.

 1. While the paper adeptly explores the role of technology in RCT recruitment through a wellexecuted dual-phase qualitative study, a noteworthy limitation lies in the small sample size, particularly in the PPI focus group where data saturation becomes challenging with only three participants. The authors acknowledge this limitation, but clarity on the rationale for this specific number is lacking.

**Response 1: Many thanks for this comment. Please see Response 1 to Reviewer 1.**

 2. Furthermore, while the recruitment process is adequately detailed, inclusion of participant

 characteristics, such as gender, experience, or roles in recruitment, would enhance transparency and generalizability.

**Response 2: These are presented in Table 2.**

 3. The paper mentions using Microsoft Word for analysis without specifying the process,

 necessitating clarification on whether any qualitative software was employed for data

 management and analysis.

**Response 3: The line “** **Microsoft Office was used” is a typo – perhaps a partial remnant of a previous version. This line has been removed from the revised version of the manuscript. NVivo 12 was used to help facilitate coding. This has been added to the manuscript.**

 4. The data management and safety plan require elaboration for a comprehensive understanding.

**Response 4: Data collection, analysis and availability are all reported. Details regarding ethics have been elaborated upon for further clarity.**

 5. The results section might benefit from a more succinct presentation of main themes and sub-themes, avoiding exhaustive detail.

**Response 5: We would argue that the great detail presented is necessary to fully encapsulate participants’ perspectives and facilitate interpretation. We hope that Figure 1 succeeds in achieving a more succinct presentation for those who prefer such an approach and that the greater detail will cater to those who prefer a more comprehensive approach.**

 6. Additionally, the initial paragraphs of the discussion seem repetitive of the results and could be condensed to emphasize key discussion points more concisely.

**Response 6: Though we recognise that there is some level of repetition, this is purposeful for two reasons: first, given that the results section is on the lengthy side, it’s useful to begin the discussion with a brief summary, as a reminder for readers; second, the summary provides an outline for the interpretation of results. Yes, findings were interpreted (to an extent) within the results section, but added nuance and further implications are presented in this discussion.**

# Reviewer report: Reviewer response for version 1

**DOI:** 10.21956/hrbopenres.15069.r37636

**Published:** 2024-01-16

**Authors:** Marie Falahee

**Affiliations:** 1 University of Birmingham, Birmingham, England, UK

Thank you for the opportunity to review this interesting manuscript describing a qualitative study of researchers’ perspectives on the use of technology to facilitate recruitment to randomized clinical trials (RCTs). This is an important area of investigation given the crucial importance of successful trial recruitment for evidence-based medicine and well-documented challenges towards achieving this.

 The manuscript is extremely well written and clearly presented, and the introduction develops a strong rationale for why this research is needed. The major weakness of this study is the small sample size, though this is appropriately noted by the authors in the discussion section in relation to the adequacy of the data. However, it could be argued that saturation is unlikely to have been achieved (for example, richer insight into the relationships between equality, diversity and inclusivity issues and sample bias might be expected) and reflection on the adequacy of the recruitment approach used in this study, perhaps balanced against what is feasible within a summer studentship research project, is needed in the discussion.

 Further detail about recruitment procedures in the method section would also be helpful. The authors aimed to recruit “researchers who had previously been involved in the recruitment process for a RCT.” Most trials involve large, multidisciplinary research teams. Were all members of the identified trials approached, or just the principal investigator? Furthermore, recruitment is often delivered by clinical research staff who may not be identifiable as part of the investigator team. Were such staff invited to participate? More details about the characteristics of those approached to take part would be helpful for the reader, particularly in relation to their professional roles (e.g. clinical, academic, methodologist, etc.). Were those contacted also invited to identify/invite others who had been involved in recruitment?

 Of those researchers who agreed to participate, it would be interesting to know (especially given the topic of this manuscript) the recruitment approach that led to their participation, if this information is available. For example, which had been invited by the research team directly to take part in the study and which contacted the research team as a result of seeing an advert on social media / via a colleague?

 Minor comments:

 The results section suggests that audio recorded interviews were transcribed and checked for accuracy. This should be noted in the method section, with details of the approach taken (e.g. by whom).

 It is commendable that the authors appear to have applied the COREQ checklist. It would be helpful to include an explicit statement to this effect in the main text of the methods, rather than in a footnote, and to provide a completed COREQ checklist as supplementary material.

 Patient and public partners could have been involved in other aspects of the research, such as the development of the qualitative interview schedule and the development of the recruitment procedures, though this is not described.

 Whilst reading the manuscript I noticed some typographical errors. For example, in Table 3: ‘frquently’ should be 'frequently'. There is erroneous repetition of ‘With’ in the second sentence of the second paragraph in the introduction.

Is the work clearly and accurately presented and does it cite the current literature?

Yes

If applicable, is the statistical analysis and its interpretation appropriate?

Not applicable

Are all the source data underlying the results available to ensure full reproducibility?

Yes

Is the study design appropriate and is the work technically sound?

Partly

Are the conclusions drawn adequately supported by the results?

Yes

Are sufficient details of methods and analysis provided to allow replication by others?

Partly

Reviewer Expertise:

Psychology, Rheumatology, Patient and Public Involvement in Research

I confirm that I have read this submission and believe that I have an appropriate level of expertise to confirm that it is of an acceptable scientific standard, however I have significant reservations, as outlined above.

# Response

**Published:** 2024-05-29

**Authors:** Christopher Dwyer

**Affiliations:** Technological University of the Shannon Midlands Midwest, Athlone, Westmeath, Ireland

Many thanks for your comments. We considered each in turn and have amended the manuscript in light of these considerations. Other comments and questions we answer here.

 1. The major weakness of this study is the small sample size, though this is appropriately noted by the authors in the discussion section in relation to the adequacy of the data. However, it could be argued that saturation is unlikely to have been achieved (for example, richer insight into the relationships between equality, diversity and inclusivity issues and sample bias might be expected) and reflection on the adequacy of the recruitment approach used in this study, perhaps balanced against what is feasible within a summer studentship research project, is needed in the discussion.

**Response 1: As indicated, we certainly acknowledge the small sample size perspective – indeed, a larger sample was desired. We also acknowledge, that saturation may not have been reached. However, if one is to subscribe to the perspective that saturation operationally refers to the observation of the same themes emerging, there is an argument that this study achieved that, at least to some extent; for example, in the cases of the five sub-themes identified (which were thematically organised into two themes and one superordinate theme) one was discussed by 5 of 7 participants (i.e.** ***Proclivity for easy and fast*** **), three by 6 of 7 participants (i.e. *Avoidance of resource-intensive methods*; *Gatekeeping & Referral*; and *Ethical Considerations*) and one by 7 of 7 participants ( *Recruitment strategy should be dictated by the cohort*). With that, t** **he limitations section has been amended to reflect such further considerations as well as the points made in the reviewer’s comment.**

 2. Further detail about recruitment procedures in the method section would also be helpful. The authors aimed to recruit “researchers who had previously been involved in the recruitment process for a RCT.” Most trials involve large, multidisciplinary research teams. Were all members of the identified trials approached, or just the principal investigator? Furthermore, recruitment is often delivered by clinical research staff who may not be identifiable as part of the investigator team. Were such staff invited to participate? More details about the characteristics of those approached to take part would be helpful for the reader, particularly in relation to their professional roles (e.g. clinical, academic, methodologist, etc.). Were those contacted also invited to identify/invite others who had been involved in recruitment?

**Response 2: The following has been added to the methodology: “** **Specifically, in each of the 50 trials, both the lead author and corresponding author (if different individuals) were identified for contact, based on the commonality of these individuals having the most input into a study’s administration. If these individuals were not involved in the recruitment process, they were asked in the email to advise who best to contact in this context (given that not all published articles include a break-down of author involvement). In the event of non-response (i.e. after two weeks, a second email was sent.** **” With that, we recognise that in cases where no response was received, contact details could have been found for other researchers from the study and an email sent querying who most appropriate to contact in light of the null response. However, such activity would have been time costly and as the reviewer correctly identified with respect to what is feasible within a summer studentship research project, such activity was not engaged.**

 3. Of those researchers who agreed to participate, it would be interesting to know (especially given the topic of this manuscript) the recruitment approach that led to their participation, if this information is available. For example, which had been invited by the research team directly to take part in the study and which contacted the research team as a result of seeing an advert on social media / via a colleague?

**Response 3: Many thanks for this comment – it is certainly an interesting and very relevant point. Unfortunately, we do not have data on this. Moreover, though it would certainly be interesting, we’re not entirely sure how much we could take from it, interpretation-wise, given it being based on only 8 occurrences (including the participant lost to scheduling). However, should future research further investigate this topic, for example, via survey – where a much larger sample size would be required, inclusion of such data collection is certainly recommended.**

 4. The results section suggests that audio recorded interviews were transcribed and checked for accuracy. This should be noted in the method section, with details of the approach taken (e.g. by whom).

**Response 4: Such discussion appears in the data analysis section of the methodology. The approach is now described and the researchers who checked for accuracy identified.**

 5. It is commendable that the authors appear to have applied the COREQ checklist. It would be helpful to include an explicit statement to this effect in the main text of the methods, rather than in a footnote, and to provide a completed COREQ checklist as supplementary material.

**Response 5: Though we have maintained the footnote, given that it facilitates sufficient detail (and would perhaps divert the logic elsewhere placed, we have included a brief note about this in the methodology as well, with the completed COREQ checklist added as an appendix.**

 6. Patient and public partners could have been involved in other aspects of the research, such as the development of the qualitative interview schedule and the development of the recruitment procedures, though this is not described.

**Response 6:** **Although we certainly see the value of engaging PPI for the development of interview schedules such as this, the focus of our rationale for the interview guide  was development in light of both findings from the PRioRiTy I study** **^5^** **and, specifically, a group of researchers experienced in the recruitment process (e.g. with respect to theory, administration and other background activities). Moreover, PPI (especially those involved in Phase 2) was excluded from interview guide development in an effort to reduce bias. With that, PPI views and perspectives certainly come through in light of their Phase 2 engagement. Likewise,** **we certainly see the value of engaging PPI for the development of recruitment procedures, but given the nature of the primary cohort (i.e. researchers) as well as time constraints associated with the study, a researcher developed recruitment protocol was utilised.**

 7. Whilst reading the manuscript I noticed some typographical errors. For example, in Table 3: ‘frquently’ should be 'frequently'. There is erroneous repetition of ‘With’ in the second sentence of the second paragraph in the introduction.

**Response 7:** **Many thanks for spotting these – they have been amended.**
